# Sonographic Evaluation of Plantar Hindfoot Pain in Athletes

**DOI:** 10.1007/s12178-026-10063-6

**Published:** 2026-10-21

**Authors:** Tyra L. Swanson, Brennan J. Boettcher, Douglas F. Hoffman, Walter I. Sussman, Ryan C. Kruse

**Affiliations:** 1 Department of Physical Medicine and Rehabilitation, Mayo Clinic, Rochester, MN USA; 2 Department of Orthopedic Surgery, Division of Sports Medicine, Mayo Clinic, Rochester, MN USA; 3 Departments of Orthopedics and Radiology, Essentia Health, Duluth, MN USA; 4 Boston Sports & Biologics, Wellesley Hills, MA USA; 5 Department of Orthopedics and Rehabilitation, Tufts Medical Center, Boston, MA USA; 6 Department of Orthopedics and Rehabilitation, University of Iowa, Iowa City, IA USA

**Keywords:** Musculoskeletal ultrasound, Plantar heel pain, Plantar fascia, Heel fat pad, Calcaneal pathology, Sports-related hindfoot injuries

## Abstract

**Purpose of Review:**

To provide an anatomy-based review of musculoskeletal ultrasound (US) in the evaluation of plantar hindfoot pain in athletes. This review summarizes normal sonographic anatomy, characteristic findings, diagnostic utility, and limitations for plantar fascia, heel fat pad, calcaneal, peripheral nerve, vascular, and foreign-body pathology. It also examines how US findings should be integrated with the history and physical examination in athletes.

**Recent Findings:**

Recent studies support high-resolution, dynamic US as a useful imaging modality for plantar hindfoot pain. US enables real-time assessment, contralateral comparison, sonopalpation, and evaluation during dynamic maneuvers. Emerging literature has further characterized the layered structure and loading behavior of the heel fat pad, dynamic sonographic findings of fat pad fracture and subluxation, and assessment of peripheral nerve entrapment. Recent studies also demonstrate that plantar fascia, heel fat pad, and other hindfoot abnormalities are prevalent in asymptomatic athletes. Although sonographic abnormalities may be associated with future time-loss injury, their low positive predictive value limits their role as a screening tool. MRI remains important when evaluating suspected calcaneal stress injury, marrow pathology, or complex soft-tissue disease.

**Summary:**

US is an accessible and valuable modality for the assessment of plantar heel pain in athletes. A structured, symptom-driven examination may improve diagnostic precision and identify alternative or coexisting pathology. Sonographic findings should be correlated with symptom location, physical examination, and sonopalpatory pain, particularly because structural abnormalities are common in asymptomatic athletes.

## Introduction

Plantar heel pain is a common and often performance-limiting complaint in athletic populations, with a broad differential diagnosis spanning osseous, soft tissue, neurologic, and systemic etiologies [1]. While plantar fasciopathy remains the most frequently implicated condition, accounting for over one million patient visits annually in the United States, athletes are uniquely susceptible to a broad spectrum of hindfoot pathologies due to repetitive loading, sport-specific biomechanics, and high training volumes [2]. Accurate and timely diagnosis is critical, as mischaracterization of the underlying pathology may lead to prolonged symptoms, inappropriate management, and delayed return to sport [2, 3].

Imaging plays an important role in obtaining an accurate diagnosis. Among available imaging modalities, musculoskeletal ultrasound (US) has emerged as an increasingly valuable tool in the assessment of plantar hindfoot pathology [4]. US offers several advantages particularly relevant to the athletic population, including dynamic real-time assessment, side-to-side comparison, portability, and cost-effectiveness [5]. Additionally, it allows for correlation of abnormal findings with sonopalpatory pain, which is not a feature of other imaging modalities. It provides high-resolution visualization of soft tissue structures such as the plantar fascia, plantar heel fat pad, and peripheral nerve branches, while also providing valuable information regarding various osseous pathologies of the calcaneus [5, 6].

This review provides a comprehensive, anatomy-based approach to the sonographic evaluation of plantar hindfoot injuries in athletes, emphasizing normal sonographic anatomy, pathologic findings, diagnostic pitfalls, and the integration of US into clinical decision-making within the unique context of the athletic population.

## Plantar Fascia

### Normal Anatomy

The plantar fascia is a dense fibrous aponeurosis originating from the medial calcaneal tubercle and serving as a primary stabilizer of the longitudinal arch [2, 7, 8]. It is composed of three distinct components: the central cord, lateral cord, and medial band. The central cord is the thickest and most functionally significant, extending distally to divide into five digital slips inserting at the metatarsophalangeal joints and blending with the dermis, flexor tendon sheaths, and transverse metatarsal ligaments. The lateral cord arises from the lateral aspect of the medial calcaneal tubercle and inserts at the lateral and plantar base of the fifth metatarsal. In contrast, the medial band is thinner, arising from the central cord and blending with the fascia overlying the abductor hallucis muscle [2, 7, 9].

### Normal Sonographic Appearance

Sonographic evaluation of the plantar fascia is typically performed with the patient prone, with the feet extending beyond the edge of the examination table [7, 10, 11]. A high-frequency linear transducer is preferred, although a medium-frequency linear transducer may be needed for patients with thick, calloused skin [11, 12]. Standard imaging includes long-axis (LAX) views of the entire extent of all fascial components, while using short-axis (SAX) views to confirm focal pathology and measure fascial thickness. Dynamic maneuvers such as passive toe dorsiflexion (windlass mechanism) are often used to evaluate fascial tension and integrity [5, 7, 12].

**Fig. 1:**
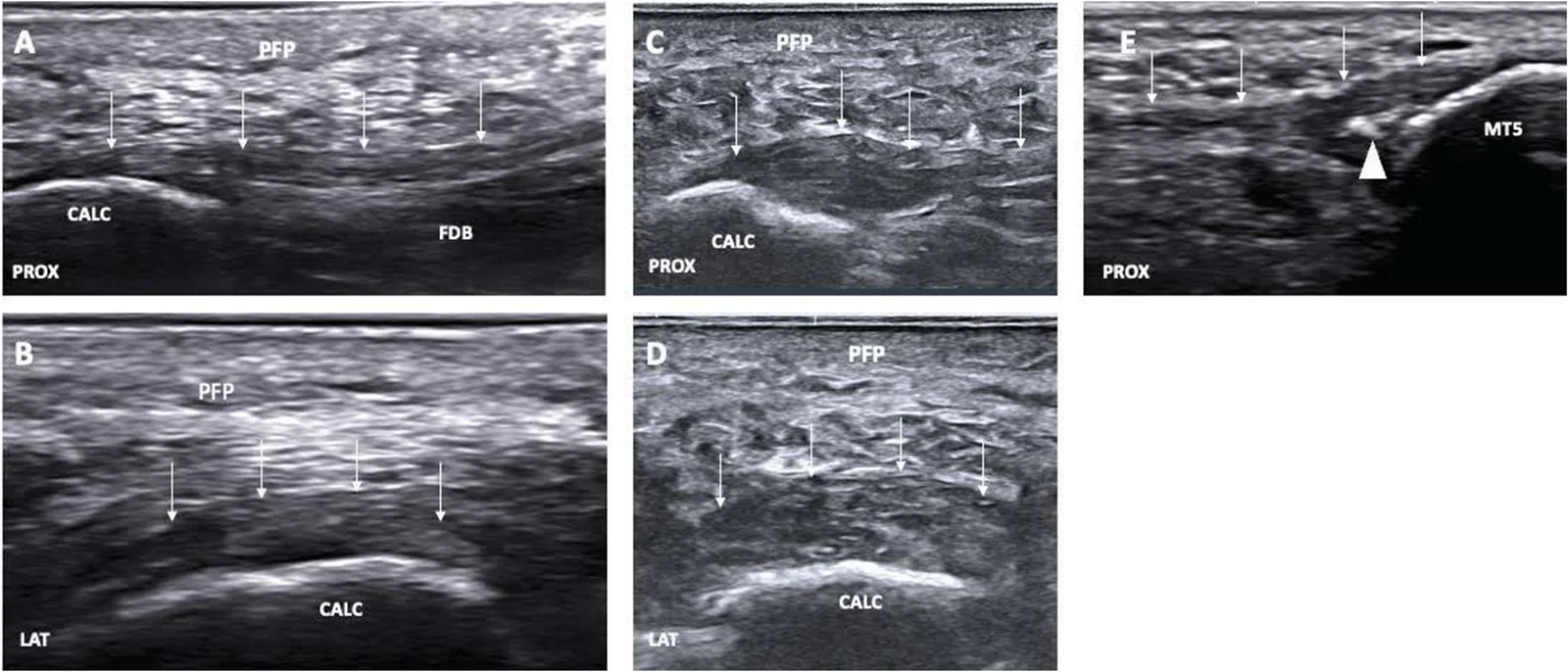
**A.** Normal long-axis (LAX) sonographic appearance of the plantar fascia. **B.** Normal short-axis (SAX) sonographic appearance of the plantar fascia. **C.** SAX and (**D**) LAX views of hypoechoic thickening of the plantar fascia, consistent with plantar fasciopathy. Distal lateral cord plantar fasciopathy at the insertion on the 5th metatarsal (MT5). Note a small intrafascial calcification (closed white arrowhead). PROX = proximal; LAT = lateral; CALC = calcaneus; PFP = plantar fat pad; FDB = flexor digitorum brevis muscle; closed white arrows = plantar fascia

Sonographically, the plantar fascia normally appears as a homogeneous, fibrillar, hyperechoic band with well-defined margins and thickness of approximately 2–4 mm at the calcaneal origin of the central cord (Fig. 1A-B) [12, 13]. The fascia should maintain a uniform echotexture without focal hypoechogenicity, fiber disruption, or hyperemia [11, 12]. Given substantial inter-individual variability, contralateral comparison can help to distinguish physiologic adaptation from pathologic change, particularly in high-load athletic populations where mild thickening or heterogeneity may be incidental rather than symptomatic [7, 14].

### Plantar Fasciopathy

Plantar fasciopathy represents the most common cause of plantar heel pain in athletic populations, with a lifetime incidence of approximately 10% and affecting up to 18% of running and jumping athletes [1, 2, 10, 15]. Biomechanical risk factors include limited ankle dorsiflexion, increased body mass index, pes planus or cavus deformities, and abrupt changes in training volume or intensity [2, 16]. Plantar fasciopathy is particularly common in runners and court-sport athletes, in whom repetitive push-off and rapid training-load progression may increase fascial loading. The pathology most commonly involves the proximal central cord at its calcaneal origin [2, 13]. Clinically, plantar fasciopathy often presents with insidious onset medial plantar heel pain, classically worst with the first steps after rest and exacerbated by prolonged weight-bearing activity [1, 2]. Physical examination (PE) findings typically include pain with palpation of the plantar fascia origin.

Plantar fascia thickness > 4 mm is commonly considered pathologic, however fascial thickness alone is not sufficient for the diagnosis of symptomatic plantar fasciopathy, particularly in athletes. Sonographic findings include hypoechogenicity, loss of normal fibrillar echotexture, cortical irregularity or enthesopathy at the calcaneal origin, fascial hyperemia, and varying degrees of partial thickness tearing (Fig. 1C-D) [4, 7, 10, 12]. Mid-substance fasciopathy may also occur, but is less common than proximal pathology (Fig. 1E) [17].

### Plantar Fascia Tear

Plantar fascia tears are uncommon but clinically significant injuries that typically occur in athletes during high-load activities such as sprinting or jumping, often superimposed on underlying fasciopathy or following an intrafascial or perifascial corticosteroid injection [8, 18]. Most frequently involved is the proximal third of the central cord, and much less likely the lateral cord or medial band. Clinically, plantar fascia tears present with acute onset plantar heel pain often accompanied by a “pop,” followed by difficulty with weight-bearing and localized swelling or ecchymosis [19, 20]. Passive dorsiflexion of the toes may reproduce pain and reveal loss of normal fascial tension [19–21].

**Fig. 2:**
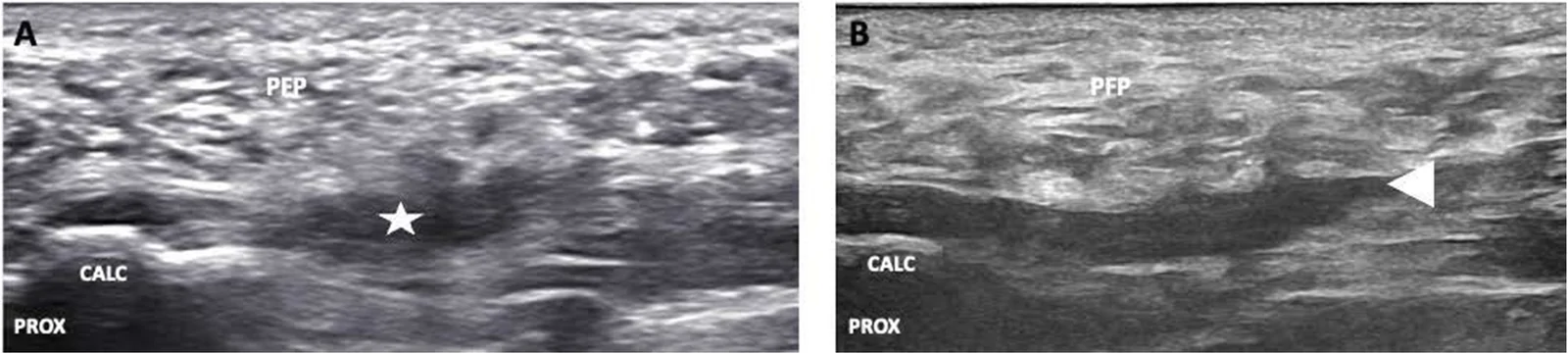
**A.** Long-axis (LAX) sonographic image of a partial thickness tear (closed white star) of the central cord plantar fascia. **B.** LAX sonographic image of a mid-substance central cord plantar fascia rupture, with loss of fascial tension. The proximal fascial stump is denoted by the closed white arrowhead. The visualized fascia is significantly fasciopathic. PROX = proximal; PFP = plantar fat pad

In plantar fascia tears, US reveals fiber disruption with a variable degree of tension loss (Fig. 2A) [4, 5, 12, 19, 21]. Partial-thickness and full-thickness but incomplete width tears demonstrate focal fiber disruption with preserved fascial continuity and tension, while full-thickness full-width (complete) tears demonstrate complete fiber discontinuity with loss of tension statically and with dynamic maneuvers (Fig. 2B) [19, 21]. Additional findings may include surrounding edema and variable retraction depending on tear severity [19, 21].

### Plantar Fibromatosis

**Fig. 3:**
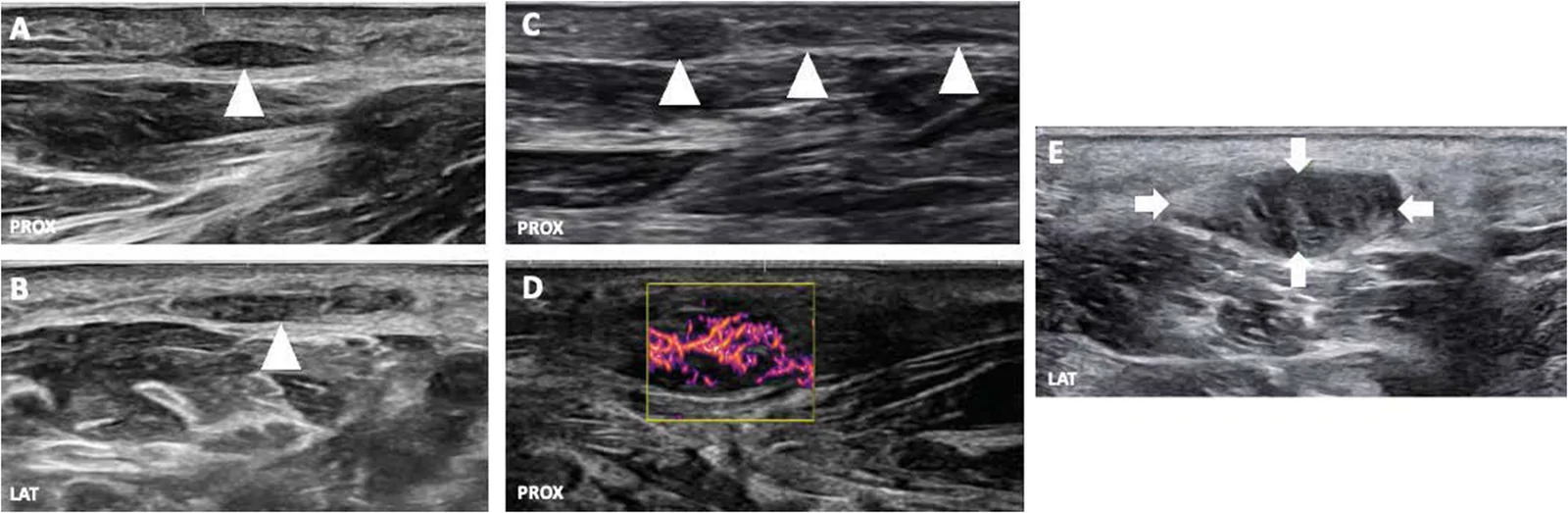
Plantar fibromatosis. **A.** Long axis (LAX) and (**B**) short axis (SAX) sonographic appearance of a single plantar fibroma (closed white arrowhead) (**C**) Sonographic image of multiple plantar fibromas in LAX. **D.** Intrafibroma hyperemia may be seen, although not always present. (E) “Comb sign”, represented by the interdigitating hypoechoic and isoechoic lines (closed white arrows), often seen in larger fibromas. PROX = proximal; LAT = lateral

Plantar fibromatosis (Ledderhose disease) is a benign fibroproliferative disorder of the plantar fascia characterized by nodular collagen deposition within the fascia [22, 23]. The etiology is not fully understood, though associated risk factors include repetitive trauma, genetic predisposition, diabetes, and alcohol use [24, 25]. The condition most commonly affects the mid-portion or distal central cord [26]. Clinically, it presents as slowly progressive, firm, palpable plantar nodules that may be asymptomatic or mildly painful [22]. Although not classically sport specific, a plantar fibroma may become symptomatic in athletes in the setting of direct shoe pressure or repetitive plantar fascia loading which can be seen with jumping athletes. On PE, these nodules are typically well-defined, firm, and fixed within the plantar fascia, often located along the medial or central arch, and may be single or multiple, occasionally bilateral, with pain primarily elicited by direct pressure [22, 24, 27, 28]. Sonographically, findings of plantar fibromatosis include discrete hypoechoic, fusiform nodules within the fascia (Fig. 3A-C) [26–28]. A recently described sonographic feature, the “comb sign,” refers to alternating bands of hypoechogenicity and isoechogenicity within the fibroma, reported in approximately 51% of lesions and more commonly seen in larger nodules (Fig. 3E) [26]. These lesions are well-defined and focal, in contrast to the poor-demarcated, diffuse thickening seen in plantar fasciopathy, and may demonstrate mild internal vascularity (Fig. 3D) [26–28]. The surrounding fascia may appear otherwise normal. If there are any atypical sonographic findings or a rapid increase in size, plantar foot sarcomas should be included in the differential diagnosis, and advanced cross sectional imaging should be obtained [29].

## Plantar Heel Fat Pad

### Normal Anatomy

The plantar heel fat pad is a specialized fibroadipose structure that serves as the primary shock absorber during weight-bearing [30–32]. The fat pad is composed of adipose tissue organized into a honeycomb-like architecture of closed-cell chambers enveloped by fibrous septa [30, 31]. It is structurally divided into two distinct layers separated by a horizontal fibrous band: a superficial microchamber layer and a deep macrochamber layer. The superficial microchamber layer is thinner and stiffer, and functions primarily to resist shear forces and anchor the overlying skin [33]. The deep macrochamber layer is thicker, comprising approximately 70% of the total fat pad thickness, and is composed of larger adipose chambers that deform under compressive loading to provide the primary cushioning function [30, 34].

### Normal Sonographic Appearance

**Fig. 4:**
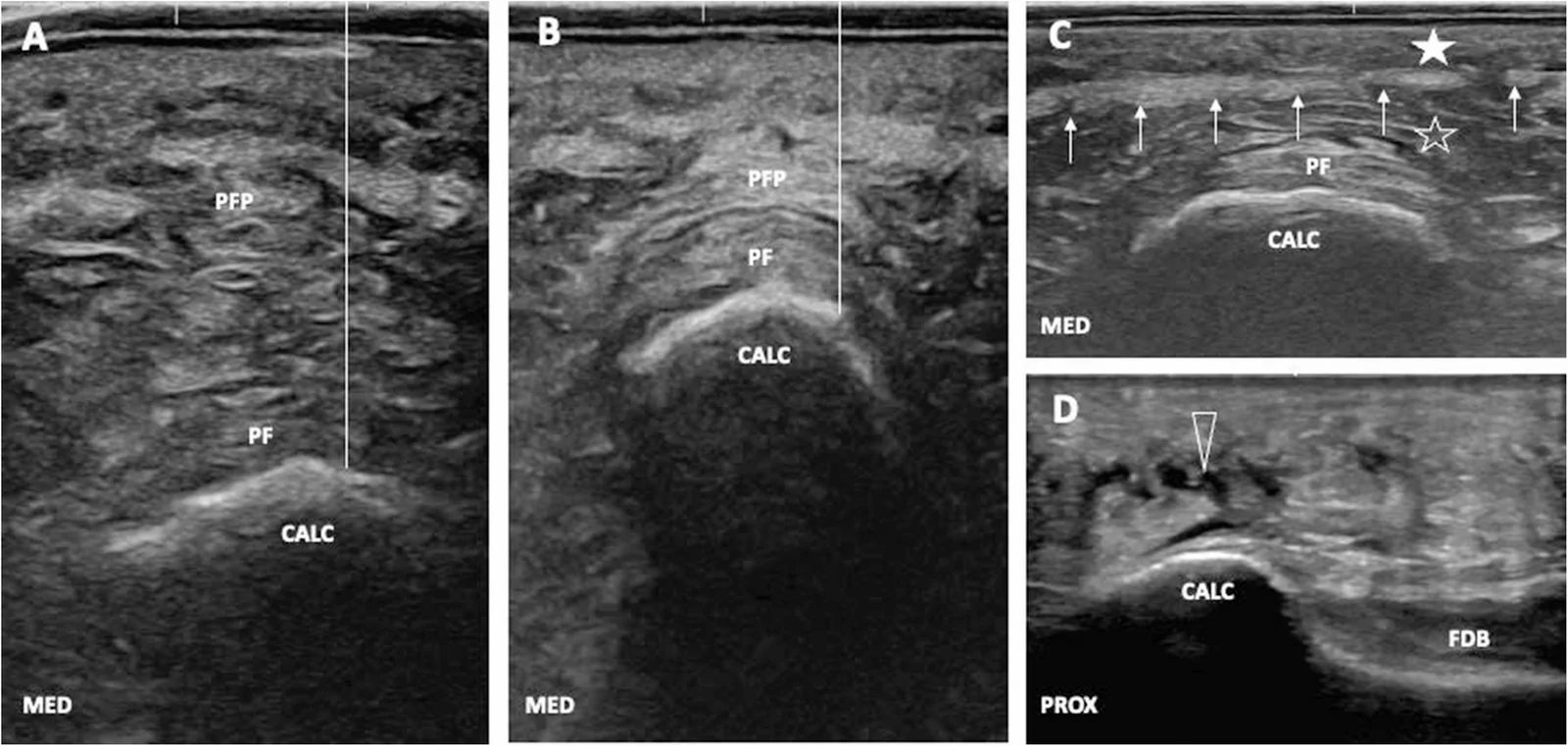
Sonographic findings of plantar fat pad abnormalities. Plantar heel fat pad insufficiency can be diagnosed sonographically by measuring the distance from the calcaneus (CALC) to the skin with no transducer pressure (**A**), and comparing that distance to an image with maximal transducer pressure (**B**). **C**. Sonographic imaging demonstrating the different components of the plantar fat pad. The superficial microchamber (closed white star) is separated from the deep macrochamber (open white star) by a thick fibrous layer (closed white arrows). **D**. An anechoic cleft within the fat pad represents a region of fat fracture (open white arrowhead). PROX = proximal; MED = medial; PF = plantar fascia; PFP = plantar fat pad; FDB = flexor digitorum brevis muscle

Sonographic evaluation of the plantar heel fat pad is performed with the patient prone, feet extending beyond the edge of the examination table, using a high-frequency linear transducer [11, 14]. Standard imaging includes LAX and SAX views centered over the inferior calcaneus, with assessment performed in both unloaded and compressed states to evaluate fat pad thickness and compressibility (Fig. 4A-B) [11, 14, 35]. The heel pad compressibility index, calculated as the ratio of compressed to uncompressed thickness, provides a functional measure of fat pad elasticity [14, 35].

Sonographically, the normal plantar heel fat pad appears as a well-organized structure with hypoechoic fat lobules separated by hyperechoic fibrous septa, maintaining a characteristic lobular echotexture (Fig. 4C) [11, 35]. The two discrete layers, superficial microchamber and deep macrochamber, can be differentiated by visualizing the echogenic horizontal fibrous band separating the chambers (Fig. 4C) [11, 34]. Additionally, the superficial microchamber appears more hyperechoic due to the increased density of fibrous septae, while the deep macrochamber has a lower density of fibrous septae and therefore appears more hypoechoic. Normal unloaded heel pad thickness is on average 14 mm in adults, with males generally demonstrating greater thickness than females [1, 14, 35]. Heel fat pad thickness and compressibility index do not appear to correlate with activity level in healthy individuals, as sonographic measurements in sedentary, moderately active, and highly active young adults have been shown to be similar [36].

### Fat Pad Insufficiency/Atrophy

Fat pad insufficiency, also referred to as heel fat pad syndrome, is a common cause of plantar heel pain resulting from degenerative or atrophic changes in the heel fat pad [1, 37, 38]. The pathophysiology involves progressive loss of adipose tissue volume as well as thinning and fragmentation of the fibrous septa, leading to inadequate cushioning of the plantar fascia and calcaneus during weight-bearing [1, 31, 32]. Risk factors include aging, prior corticosteroid injections, diabetes mellitus, increased body mass index, and repetitive high-impact loading. Hormonal changes during pregnancy may also predispose to fat pad insufficiency, with foot pain reported in up to 31% of postpartum women [39]. In athletes, fat pad insufficiency may be most symptomatic during repetitive impact activity or training on hard surfaces. Clinically, fat pad insufficiency presents as deep, bruise-like pain localized to the central or posterior heel. Pain is characteristically exacerbated by walking barefoot, on hard surfaces, or for prolonged periods, typically without pain during the first few steps in the morning that is typically seen with plantar fasciopathy [1, 37]. PE may reveal visible loss of heel pad bulk, with the calcaneus often palpable through thinned subcutaneous tissue.

On US, an atrophic or insufficient fat pad demonstrates decreased fat pad thickness, loss of the normal lobular architecture, thinning or fragmentation of the fibrous septa, and increased echogenicity of the fat pad reflecting fibrosis. Compressibility index assessment may reveal abnormally increased compressibility (> 0.60), reflecting fat pad hypercompressibility and reduced shock-absorbing capacity [14, 40]. Dysconjugate motion/separation of the adipose structure may be observed with compression. Contralateral comparison is valuable in distinguishing pathologic atrophy from physiologic variation, particularly in older adults and athletes.

### Fat Pad Contusion/Fracture

Fat pad contusion and fracture are typically seen with acute traumatic injuries to the plantar heel fat pad, often resulting from direct impact such as a fall from height or repetitive high-impact loading in running/jumping athletes [33, 37, 41]. The pathophysiology involves disruption of the fibrous septa and hemorrhagic infiltration of the adipose chambers, with contusion representing a milder injury characterized by edema and hemorrhage within intact compartments [30, 33, 41]. A recently described entity, subluxing fractured fat pad, involves dynamic displacement of the disrupted fat pad tissue during weight-bearing, with patients reporting a characteristic “snapping” or “popping” sensation in the heel [38]. Clinically, fat pad contusion may present with acute pain after a traumatic event but may also be associated with chronic degenerative changes with localized tenderness and possible ecchymosis over the central or posterior heel. Pain is exacerbated by direct pressure and weight-bearing on hard surfaces. Fat pad fracture may present similarly but with more severe pain, difficulty with weight-bearing, and the aforementioned mechanical symptoms. PE reveals tenderness to direct palpation over the central heel, and visible swelling or ecchymosis may be present [33, 38, 41].

Sonographically, fat pad contusions demonstrate loss of the normal lobular echotexture, while the fibrous septa may appear thickened or indistinct [30, 41]. Fat pad fracture shows more extensive disruption, with loss of the normal lobular pattern, focal and distinct tearing of the hyperechoic fibrous septa, and intervening hypoechoic to anechoic edema replacing the normal compartmentalized architecture (Fig. 4D) [30, 33, 41]. In a subluxing fractured fat pad, dynamic US is particularly valuable, demonstrating displacement or subluxation of the disrupted fat pad tissue with manual compression, a finding that may be missed on static MRI [33, 41].

## Calcaneal Pathology

### Normal Anatomy

The calcaneus is the largest tarsal bone and the primary weight-bearing structure of the hindfoot, sustaining approximately 50% of the body’s weight-bearing load during ambulation [42]. The medial plantar process serves as the origin of the plantar fascia and intrinsic foot musculature, while the posterior tuberosity provides the insertion for the Achilles tendon [43].

### Normal Sonographic Appearance

Sonographic evaluation of the calcaneus is performed with the patient in the prone position with the feet extending beyond the edge of the examination table, using a high-frequency linear transducer. Standard imaging includes LAX and SAX views along the plantar, posterior, medial, and lateral calcaneal surfaces, with assessment of the cortical contour, periosteum, and overlying soft tissues [44, 45].

**Fig. 5:**
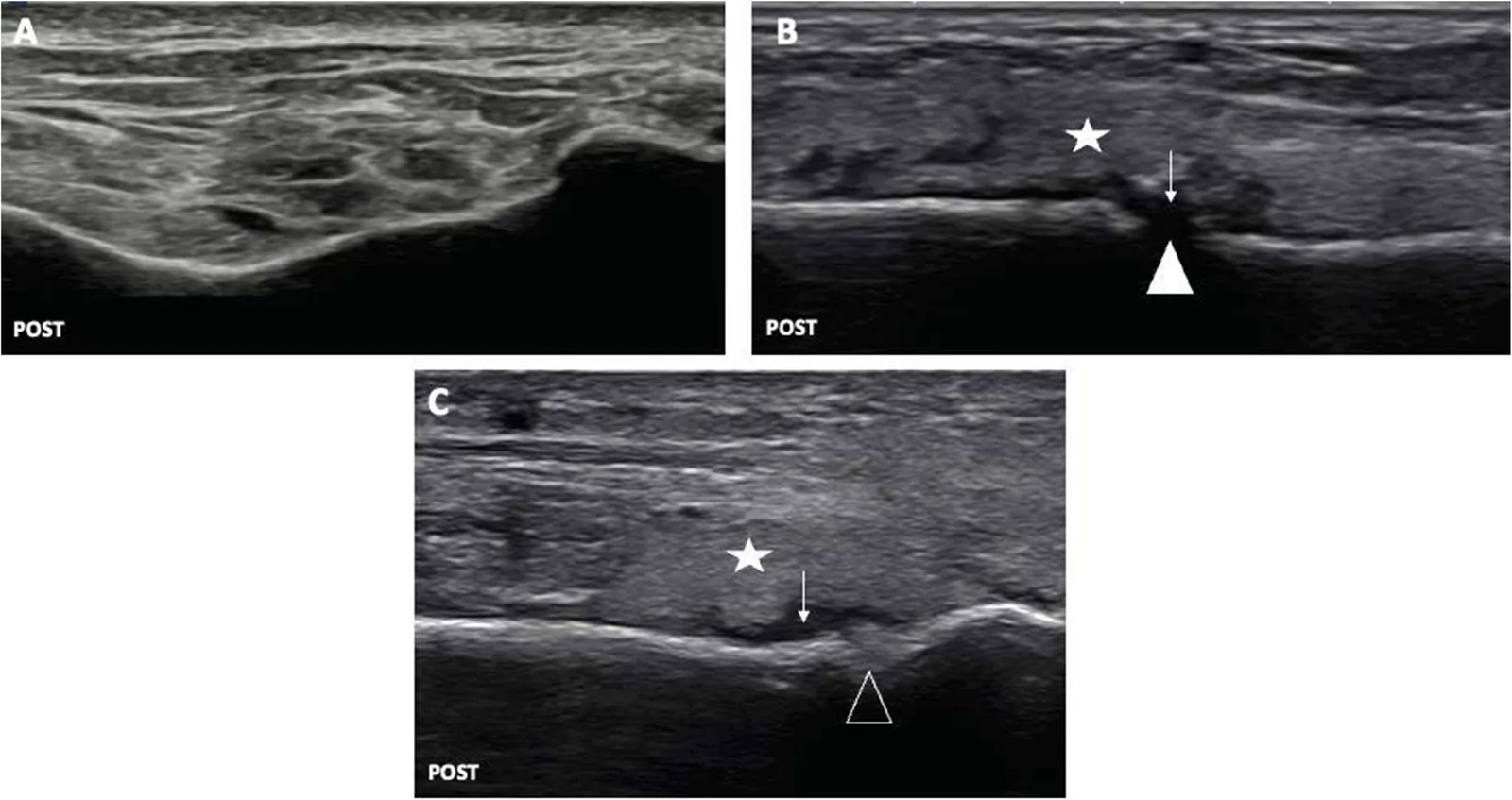
**A.** Normal appearance of the calcaneus, with a continuous, hyperechoic cortex. **B.** Subacute fracture of the calcaneus, with a cortical break (closed white arrowhead) and overlying periosteal (white arrow) and subcutaneous edema (closed white star). **C.** Calcaneal stress injury. There is cortical irregularity of the cortex (open white arrowhead) without cortical break. There is overlying periosteal and subcutaneous edema as well. POST = posterior

The calcaneal cortex normally appears as a smooth, continuous, hyperechoic line with posterior acoustic shadowing, and the overlying periosteum is seen as a thin, echogenic layer (Fig. 5A) [44, 45]. Doppler evaluation is performed to evaluate for periosteal hypervascularity, which may indicate underlying osseous pathology [45]. Contralateral comparison helps distinguishing subtle cortical irregularities from normal anatomic variation [5]. Notably, US cannot evaluate subcortical bone or bone marrow, and therefore trabecular stress injuries without cortical involvement may be missed on sonographic examination [46].

### Calcaneal Stress Injury

Calcaneal stress injuries represent a spectrum of overuse-related bone pathology ranging from stress reaction (grades 1–3) to frank stress fracture (grade 4) [47]. The calcaneus is the second most common site of stress fracture in the foot, with injuries most commonly occurring inferior and posterior to the posterior facet of the subtalar joint [10]. Risk factors include abrupt increases in training volume or intensity, low energy availability and features of Relative Energy Deficiency in Sport (REDs), menstrual dysfunction, and low bone mineral density [48, 49]. These injuries should be considered in runners, gymnasts, volleyball players, and other athletes with repetitive impact loading, particularly after a rapid change in training volume or surface. Clinically, calcaneal stress injuries present with gradually progressive activity-related heel pain that may advance to pain at rest as the injury progresses. A positive calcaneal squeeze test suggests the diagnosis, and difficulty performing a single-leg hop test provides further diagnostic support [10, 47].

Sonographically, calcaneal stress injuries may demonstrate a constellation of findings, including periosteal thickening, subcutaneous edema, and periosteal hypervascularity (Fig. 5C) [45]. Although these findings are nonspecific in isolation, they may prompt further evaluation with MRI, which remains the preferred imaging modality. Because US cannot evaluate subcortical bone, trabecular stress injuries without cortical involvement represent a key limitation of sonographic assessment [34, 47].

### Calcaneal Fracture

Acute calcaneal fractures typically result from high-energy axial loading, most commonly falls from height or motor vehicle collisions, but can also occur in high-energy landing in jumping athletes. Most occur in younger, working-age men, with a clinical presentation of a painful plantar heel with inability to bear weight, and bruising extending into the arch (Mondor sign) [50]. PE reveals marked tenderness, loss of heel height and widening of the heel, and potential compromise of the surrounding soft tissues [50].

Sonographically, acute calcaneal fractures may be identified as cortical irregularities or step-offs along the calcaneal surface (Fig. 5B) [5]. However, US cannot evaluate subcortical bone or the full extent of the fracture pattern, and therefore cannot replace CT for fracture characterization and surgical planning [46, 47]. The primary role of sonography is as an initial point-of-care tool when radiography is unavailable or as an incidental finding during evaluation for suspected soft tissue pathology [5, 45].

## Nerve-Related Pathology

### Normal Anatomy

Peripheral nerves are composed of individual axons grouped into fascicles, each surrounded by perineurium, with the fascicles collectively enclosed by the epineurium. Peripheral nerves are susceptible to injury at sites of anatomic constraint, often where they traverse fibro-osseous tunnels, pass between muscle planes, or course adjacent to bony prominences [51, 52].

### Normal Sonographic Appearance

**Fig. 6:**
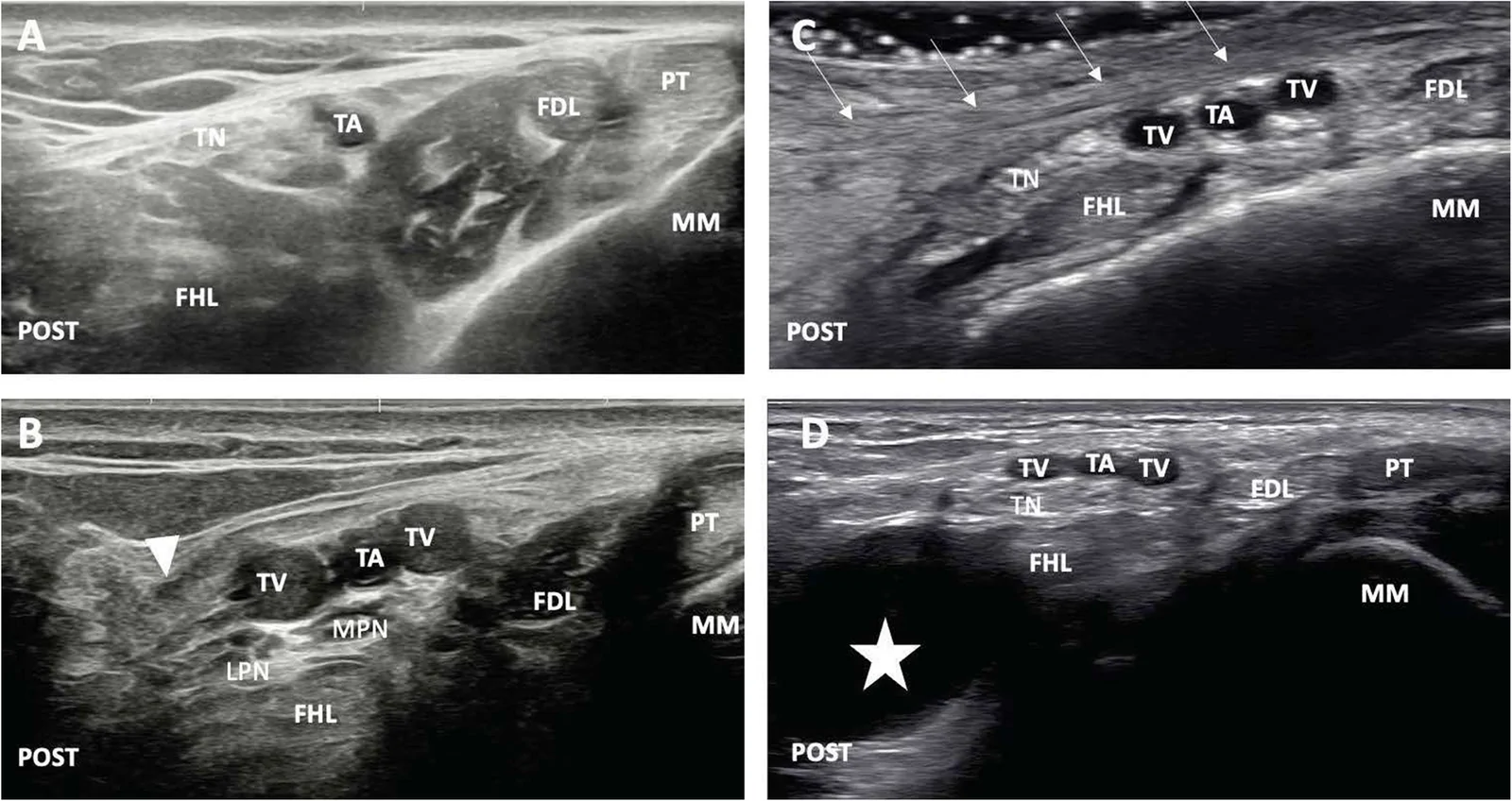
Sonographic appearance of the tibial nerve (TN) in the tarsal tunnel. **A.** Normal, short-axis (SAX) sonographic image of the tibial nerve. **B.** Tibial nerve trifurcation at the distal tarsal tunnel into the medial plantar nerve (MPN), lateral plantar nerve (LPN), and medial calcaneal nerve (closed white arrowhead). **C.** Tibial nerve compression between the flexor hallucis longus (FHL) tendon and flexor retinaculum (closed white arrows). **D.** The tibial nerve may also be compressed in the tarsal tunnel by space occupying lesions, such as a large FHL tendon sheath ganglion cyst (closed star). POST = posterior; TA = tibial artery; TV = tibial vein; FDL = flexor digitorum longus tendon; PT = posterior tibialis tendon; MM = medial malleolus

Sonographic evaluation of peripheral nerves is performed using a high-frequency linear transducer, imaging the nerve in both SAX and LAX along their course [6, 44]. In SAX, a normal peripheral nerve demonstrates a characteristic honeycomb appearance, with hypoechoic fascicles outlined by hyperechoic perineurial rims and enclosed within a hyperechoic epineurium (Fig. 6A-B). In LAX, the nerve appears as a tubular structure with alternating hypoechoic and hyperechoic parallel lines, corresponding to fascicles and connective tissue interfaces. Their sonographic appearance can be distinguished from tendons by the less compact, less uniformly echogenic internal architecture [52].

### Tarsal Tunnel Syndrome

Tarsal tunnel syndrome (TTS) is a compressive neuropathy of the tibial nerve within the tarsal tunnel [1, 53]. The exact cause is unidentified in 20%-40% of cases, with common etiologies including trauma, compressive lesions (ganglion cysts, tenosynovitis, tumors, varicosities), systemic disease-causing soft tissue edema (rheumatoid arthritis, diabetes), and abnormal biomechanics such as excessive pronation [53, 54]. In athletes, repetitive ankle motion, prior ankle trauma, and sport-specific footwear may contribute to dynamic or exertional symptoms. TTS classically presents with pain and/or neuropathic symptoms at the medial ankle and plantar foot. On examination, pain may be reproduced by tapping along the course of the nerve (Tinel sign) and with provocative maneuvers such as the dorsiflexion-eversion test. In severe cases, muscle atrophy may be observed [1, 10].

The primary sonographic finding in TTS is focal compression or increased tibial nerve cross-sectional area (CSA) at the level of the tarsal tunnel, as well as variable degrees of fascicular loss (Fig. 6C) [55]. A meta-analysis of normative data established a mean tibial nerve CSA of 10.9 mm² at the medial malleolus, with a commonly used pathologic cutoff of 11.8 mm² [56]. However, this absolute threshold has moderate sensitivity, and the within-tunnel-to-proximal-tunnel CSA ratio (cutoff > 1) may be a more accurate diagnostic parameter [55]. US may also identify a compressive etiology in approximately 50%-80% of cases (Fig. 6D) [5]. US may remain unremarkable in approximately one-third of patients with electrophysiologically confirmed TTS, representing a key limitation [55].

### Inferior Calcaneal Neuropathy (Baxter’s Neuropathy)

Compressive neuropathy of the inferior calcaneal nerve (ICN), often called Baxter’s nerve or referred to as the first branch of the lateral plantar nerve, accounts for up to 20% of chronic heel pain cases [3, 57]. The ICN is a mixed sensorimotor nerve providing sensory fibers to the long plantar ligament and calcaneal periosteum, and motor innervation to the abductor digiti minimi (ADM) muscle, and occasionally the flexor digitorum brevis (FDB) and quadratus plantae (QP) muscles [58]. Common entrapment sites include proximally between the abductor hallucis (AH) and QP muscles, and distally between the medial calcaneal tuberosity and FDB muscle [53, 58]. Clinically, ICN entrapment presents with medial plantar heel pain that is frequently misdiagnosed as plantar fasciopathy. Symptoms may be provoked by repetitive push-off activities and can be particularly limiting in running and court sports. On PE, the point of maximal tenderness is over the medial heel cephalad to the plantar fascia origin, and in severe cases lateral foot atrophy and fifth-digit abduction weakness may be observed [53].

**Fig. 7:**
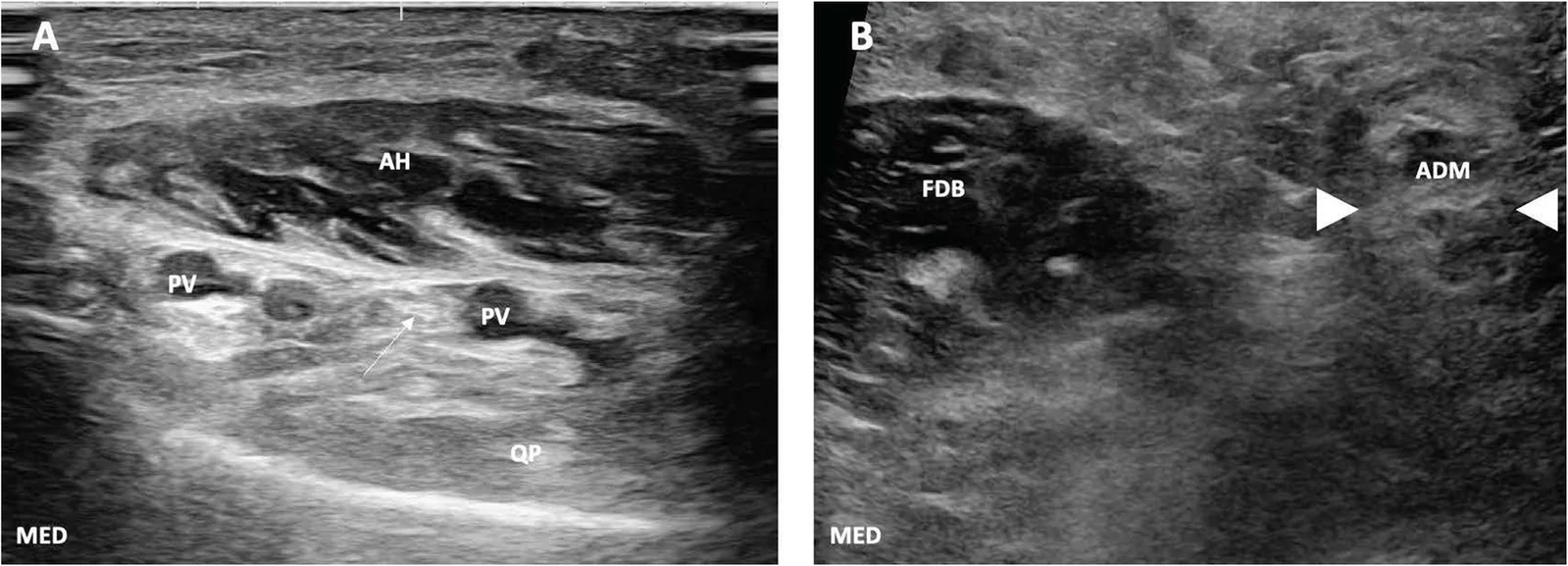
**A.** Sonographic appearance of the inferior calcaneal nerve (closed white arrow) which can be visualized between the abductor hallucis muscle (AH) and quadratus plantae muscle (QP). **B.** In compressive neuropathies of the inferior calcaneal nerve, the abductor digiti minimi muscle (ADM) may demonstrate denervation changes and fatty atrophy (region between closed white arrowheads). Compare the sonographic appearance of the denervated ADM to the adjacent, normal-appearing flexor digitorum brevis muscle (FDB) MED = medial; PV = plantar vein

Sonographically, the ICN is best visualized as it branches from the LPN at the distal tarsal tunnel or between the QP and AH muscles, though its small caliber and variable anatomy can make consistent identification challenging (Fig. 7A). US allows assessment of surrounding structures that may contribute to entrapment, including calcaneal enthesophytes, plantar fascial thickening, and varicosities [5, 59]. Indirect findings of chronic ICN entrapment include denervation changes of the ADM, appreciated as decreased muscle bulk and hyperechogenicity, though these findings can be found in approximately 4–11% of asymptomatic subjects (Fig. 7B) [5, 57, 59].

## Less Common Considerations

### Plantar Vein Thrombosis

**Fig. 8:**
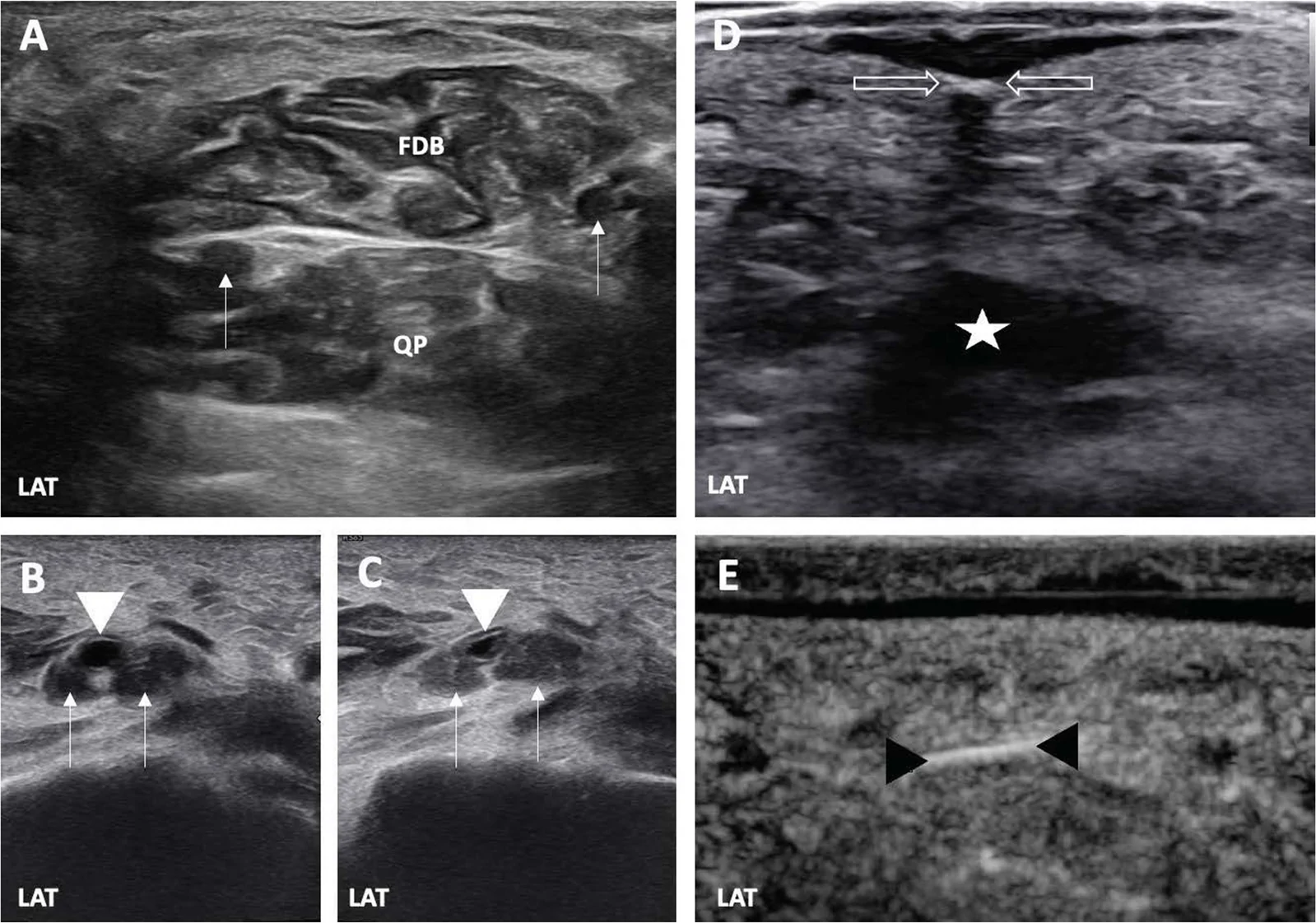
**A.** Sonographic appearance of normal, patent, medial and lateral plantar veins (closed white arrows). **B.** Sonographic appearance of the plantar veins and artery (closed white arrowhead) with no transducer pressure. Both veins and the artery appear patent. **C.** With maximal transducer pressure, the artery collapses but the veins remain non-compressible, confirming a diagnosis of plantar vein thrombosis. **D.** Subcutaneous glass foreign body (between open white arrows). Note the deep abscess (closed white star) as well as posterior acoustic shadowing. **E.** Organic foreign bodies such as wood (between closed black arrowheads) typically do not exhibit the posterior acoustic shadowing that glass foreign bodies do. LAT = lateral; FDB = flexor digitorum brevis muscle; QP = quadratus plantae muscle

Plantar vein thrombosis (PVT) is a rare but increasingly recognized mimicker of plantar fasciopathy, particularly in athletes subjected to repetitive microtrauma or prolonged weight-bearing activity [60, 61]. The condition arises from thrombosis of the lateral or medial plantar veins and may be precipitated by local venous stasis, direct mechanical trauma, or an underlying hypercoagulable state [60]. Clinically, patients present with acute plantar foot pain that is often clinically indistinguishable from a plantar fascia tear, leading to frequent misdiagnoses, though swelling is also typically present [60, 61]. US is the diagnostic modality of choice, revealing noncompressible hypoechoic or isoechoic plantar veins, with absence of flow on Doppler imaging and variable surrounding soft tissue edema (Fig. 8A-C) [62].

### Foreign Bodies

Retained foreign bodies represent an important cause of chronic, localized plantar heel pain, particularly in barefoot or outdoor athletes who sustain penetrating trauma from glass, wood, or metal fragments. US is highly sensitive for detecting both radiopaque and radiolucent foreign bodies, the latter of which, such as wood splinters or thorns, are frequently missed on conventional radiography [63]. Sonographically, foreign bodies typically appear hyperechoic on US with some degree of posterior acoustic shadowing, although not always seen with radiolucent foreign bodies. A hyperechoic, hyperemic rim may surround the foreign body, representing an inflammatory reaction that can develop within 24 h (Fig. 8D-E) [64].

## Key Consideration: Asymptomatic Sonographic Findings in Athletes

A growing body of evidence demonstrates that sonographic abnormalities of the plantar hindfoot are highly prevalent in asymptomatic athletes, reflecting physiologic adaptation rather than true pathology. In a cohort of Division I collegiate gymnasts, the majority demonstrated at least one sonographic abnormality of the hindfoot despite being entirely asymptomatic, a finding corroborated by data from a large multi-sport collegiate cohort showing a high prevalence of plantar fascia abnormalities across a variety of athletic disciplines [65–67]. These findings carry important implications for clinical practice: sonographic abnormalities must always be interpreted within the context of the patient’s symptoms, PE findings, and sonopalpatory pain, as imaging alone is insufficient for diagnosis. Additionally, contralateral comparison and integration of clinical findings remain essential to distinguish adaptive tissue remodeling from clinically significant pathology in this population [2, 7].

## Diagnostic Approach and Clinical Integration

**Fig. 9:**
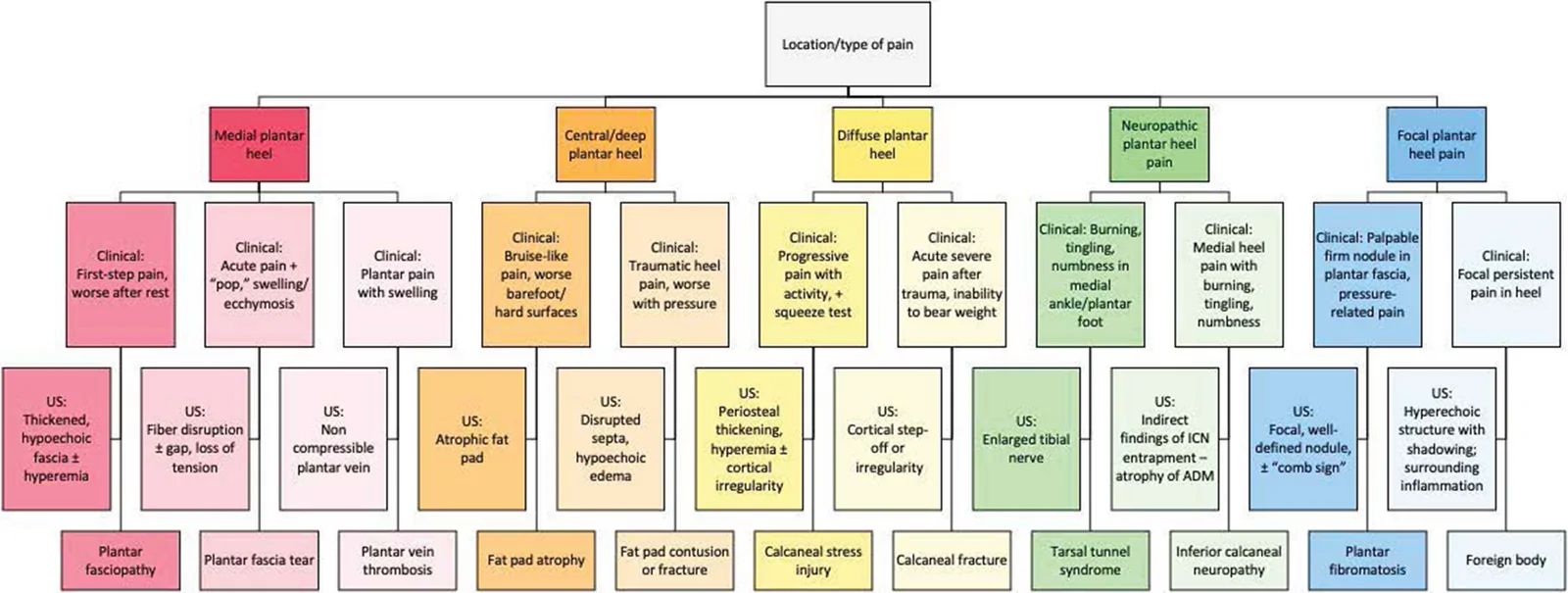
Flow diagram outlining the evaluation and diagnosis of plantar hindfoot pain in athletes, highlighting key points from the clinical history and ultrasound (US) evaluation

Evaluation of plantar hindfoot pain in athletes should follow a symptom-driven, anatomy-based approach in which history and PE guide diagnosis, with US used as a targeted adjunct rather than a primary determinant (Fig. 9). Pain location and clinical pattern remain the most useful discriminators: medial plantar heel pain with first-step symptoms suggests plantar fasciopathy, acute pain with a “pop” indicates possible fascia tear, central bruise-like pain localizes to fat pad pathology, progressive activity-related pain raises concern for calcaneal stress injury, and neuropathic symptoms suggest nerve entrapment (Table 1). US is then used to confirm the suspected diagnosis, assess severity, and identify alternative or coexisting pathology through a structured, protocol-driven evaluation. Critically, sonographic findings must be interpreted in clinical context given the high prevalence of asymptomatic abnormalities in athletes.

**Table 1:** Clinical and sonographic features of common plantar hindfoot pathologies

Pathology	Typical Clinical Presentation	Key Sonographic Findings	Distinguishing Features / Pearls
Plantar Fasciopathy	Medial plantar heel pain; worst with first steps after rest; improves then worsens with activity	Thickened (> 4 mm), hypoechoic fascia; loss of fibrillar pattern; ± hyperemia	Most common cause; correlate with symptoms due to high prevalence of asymptomatic US pathology
Plantar Fascia Tear	Acute pain with “pop”; swelling, ecchymosis; difficulty weight-bearing	Partial or complete fiber disruption; hypoechoic/anechoic gap; loss of tension	Dynamic US shows widening with dorsiflexion; often with background of fasciopathy or injection
Plantar Fibromatosis	Palpable firm nodule in arch; mild or pressure-related pain	Focal, well-defined hypoechoic fusiform nodule; ± “comb sign”	Distal/mid-fascia involvement; differentiates from diffuse proximal thickening of fasciopathy
Fat Pad Atrophy / Insufficiency	Deep, central “bruise-like” heel pain; worse barefoot/hard surfaces	Decreased thickness; loss of lobular architecture; increased echogenicity	Pain localized centrally (not medial tubercle); often in older or post-injection patients
Fat Pad Contusion / Fracture	Acute traumatic heel pain; possible ecchymosis; worse with pressure	Hypoechoic edema; disrupted septa; loss of normal architecture	Dynamic US may show displacement in “subluxing” fat pad injuries
Calcaneal Stress Injury	Progressive activity-related heel pain; + calcaneal squeeze test	Periosteal thickening; hyperemia; ± cortical irregularity	US limited – normal study does not exclude; MRI often required
Calcaneal Fracture	Acute severe pain after trauma; inability to bear weight; swelling/ecchymosis	Cortical step-off or irregularity	US adjunct only; CT required for full characterization
Tarsal Tunnel Syndrome	Burning, tingling, numbness in medial ankle/plantar foot; worse with activity	Enlarged tibial nerve (↑ CSA); possible compressive lesion	Symptoms often radiating; US may identify underlying cause (cyst, tenosynovitis)
Inferior Calcaneal Neuropathy	Medial heel neuropathic pain; may mimic fasciopathy; possible weakness	Difficult nerve visualization; indirect signs (ADM atrophy, surrounding pathology)	Consider when fasciopathy treatment fails; MRI better for denervation changes
Plantar Vein Thrombosis	Plantar pain with swelling; often mistaken for fasciopathy	Noncompressible tubular structure; absent Doppler flow	Rare but important mimic; evaluate with compression + Doppler
Foreign Body	Focal persistent pain; history may be remote or unclear	Hyperechoic structure with shadowing; surrounding inflammation	US highly sensitive, especially for radiolucent objects

*US* ultrasound, *MRI* magnetic resonance imaging, *CT* computed tomography, *CSA*  cross-sectional area, *ADM* abductor digiti minimi, *RA* rheumatoid arthritis

## Conclusion

US is a valuable adjunct in the evaluation of plantar hindfoot pain in athletes, providing high-resolution, dynamic assessment of the plantar fascia, heel fat pad, calcaneus, and peripheral nerves while facilitating timely diagnosis and targeted management. A structured, anatomy-based approach that integrates sonographic findings with the clinical history and PE is essential to distinguish pathologic conditions from common adaptive imaging findings in athletes, optimize diagnostic accuracy, and guide appropriate clinical management.

## Key References

- Expert Panel on Musculoskeletal Imaging, Said N, Fox MG, Nacey NC, Aslam F, Avery R, et al. ACR Appropriateness Criteria(R) Chronic Foot Pain: Update 2025. J Am Coll Radiol. 2026;23(4):681–99.
  - ○ This recent guideline defines the contemporary role of ultrasound within the diagnostic imaging algorithm for chronic foot pain. It supports an evidence-based approach to selecting ultrasound, MRI, radiography, or CT according to the suspected pathology.
- Morancie NA, Irvin L, Rayala BZ. Heel Pain: Diagnosis and Management. Am Fam Physician. 2025;112(6):648–56.
  - ○ This clinical review provides a practical diagnostic framework for heel pain, including key historical and examination features that differentiate plantar fasciopathy, plantar fascia tear, heel fat pad pathology, calcaneal stress injury, and nerve entrapment. It is important to recent literature because it reinforces a symptom- and examination-driven approach to imaging, supporting the central premise of this review that ultrasound findings should complement, not replace, clinical assessment.
- Holtz BO, Ceanga M, Behnert A, Marquardt R, Geis C, Axer H. Nerve Ultrasound for the Diagnosis of Tarsal Tunnel Syndrome: Findings in 26 Clinically and Electrophysiologically Confirmed Feet. J Clin Med. 2026;15(5).
  - ○ This study advances the ultrasound literature for tarsal tunnel syndrome by evaluating tibial nerve morphology in clinically and electrophysiologically confirmed cases, supporting ultrasound as an adjunctive diagnostic tool.
- Cushman DM, Carefoot A, Corcoran B, Vu L, Fredericson M, Fausett C, et al. Prevalence of Sonographic Achilles Tendon, Patellar Tendon, and Plantar Fascia Abnormalities in Division I Collegiate Athletes From a Variety of Sports. Clin J Sport Med. 2024;34(3):297–303.
  - ○ This study provides recent athlete-specific prevalence data for sonographic tendon and plantar fascia abnormalities. It reinforces the need to distinguish adaptive structural findings from clinically meaningful pathology in high-load athletes.
- Cushman DM, Stokes DC, Johnson L, Teramoto M. Prevalence of Sonographic Achilles Tendon, Patellar Tendon, and Plantar Fascia Abnormalities Among 533 Division I Collegiate Athletes from a Variety of Sports. Clin J Sport Med. 2026.
  - ○ This larger recent cohort adds to the growing literature on the frequency of baseline sonographic abnormalities in collegiate athletes. Its findings support the need for athlete-specific normative data and clinical correlation when interpreting ultrasound findings.
- Kruse RC, Rudolph L, Negaard M. Asymptomatic sonographic abnormalities of the hindfoot region in Division I collegiate gymnasts. PM R. 2024;16(6):563–9.
  - ○ This study is particularly relevant because it documents the high prevalence of asymptomatic sonographic abnormalities involving the plantar fascia, heel fat pad, and hindfoot in collegiate gymnasts. It supports the central conclusion that ultrasound findings alone should not establish the diagnosis or direct management.

## Data Availability

No datasets were generated or analysed during the current study.
